# Improved Completion Rates and Characterization of Drug Reactions with an Intensive Chagas Disease Treatment Program in Rural Bolivia

**DOI:** 10.1371/journal.pntd.0002407

**Published:** 2013-09-19

**Authors:** Jeffrey A. Tornheim, Daniel F. Lozano Beltran, Robert H. Gilman, Mario Castellon, Marco A. Solano Mercado, Walter Sullca, Faustino Torrico, Caryn Bern

**Affiliations:** 1 Combined Internal Medicine/Pediatrics Residency Program, Yale School of Medicine, New Haven, Connecticut, United States of America; 2 CEADES Salud y Medio Ambiente, Universidad Mayor de San Simon, Cochabamba, Bolivia; 3 Bloomberg School of Public Health, Johns Hopkins University, Baltimore, Maryland, United States of America; 4 Hospital Dr. Manuel Ascencio Villarroel de Punata, Punata, Bolivia; 5 Division of Epidemiology and Biostatistics, University of California San Francisco School of Medicine, San Francisco, California, United States of America; Emory University, United States of America

## Abstract

**Background:**

Chagas disease treatment is limited by drug availability, adverse side effect profiles of available medications, and poor adherence.

**Methods:**

Adult Chagas disease patients initiating 60-days of benznidazole were randomized to weekly or twice-weekly evaluations of medication adherence and screening for adverse drug events (ADEs). Mid-week evaluations employed phone-based evaluations. Adherence was measured by self-report, pill counts with intentional over-distribution, and Medication Event Monitoring Systems (MEMS). Prospective data were compared to historical controls treated with benznidazole at the same hospital.

**Results:**

162 prospective patients were compared to 172 historical patients. Pill counts correlated well with MEMS data (R = 0.498 for 7-day intervals, R = 0.872 for intervals >7 days). Treatment completion rates were higher among prospective than historical patients (82.1% vs. 65.1%), primarily due to lower abandonment rates. Rates of ADEs were lower among prospective than historical patients (56.8% vs. 66.9%). Twice-weekly evaluations increased identification of mild ADEs, prompting higher suspension rates than weekly evaluations. While twice-weekly evaluations identified ADEs earlier, they did not reduce incidence of moderate or severe ADEs. Many dermatologic ADEs were moderately severe upon presentation (35.6%), were not reduced by use of antihistamines, occurred among adult patients of all ages, and occurred throughout treatment, rather than the first few weeks alone.

**Conclusions:**

Intensive management improved completion and identified more ADEs, but did not reduce moderate or severe ADEs. Risk of dermatologic ADEs cannot be reduced by selecting younger adults or monitoring only during the first few weeks of treatment. Pill counts and phone-based encounters are reliable tools for treatment programming in rural Bolivia.

## Author Summary

Treatment of Chagas disease is complicated by poor adherence and frequent adverse drug events (ADEs). To determine the utility of pill counts and frequent monitoring to improve treatment completion and reduce ADEs, this study randomized Bolivian adults with chronic Chagas disease to either weekly or twice-weekly clinical evaluations during treatment with 60 days of benznidazole. Patient outcomes were compared to a historical cohort of patients previously treated at the same hospital with less-rigorous follow-up. This study demonstrated associations between increased follow-up and improved treatment completion, reduced ADEs, earlier documentation of ADEs, and pretreatment factors associated with benznidazole toxicity. It also validated pill-counts and demonstrated feasibility of phone-based clinical evaluations among rural Bolivians.

## Introduction

Over 100 years after its discovery, there are still few effective treatment options for Chagas disease. Chagas disease is caused by a vector-borne parasite, *Trypanosoma cruzi*, and is responsible for an estimated 430,000 DALYs lost and a cost of $1.2 billion in lost productivity each year [1]. Approximately 30% of infected adults eventually develop cardiac disease, but treatment may slow disease progression and decrease mortality [2]–[4]. Bolivia hosts disproportionately high prevalence rates and the greatest burden of cardiomyopathy and megacolon [5]. Fortunately, expanded vector control since 2000 has dramatically reduced infestation rates, opening the door for antiparasitic treatment.

Current evidence supports treatment of adults without advanced cardiac disease or significant comorbidity using either benznidazole or nifurtimox [6], [7]. Nifurtimox is associated with gastrointestinal and neuropsychiatric side effects in nearly all patients, only half of whom can tolerate the full course [8]. Benznidazole is better-tolerated, but between intermittent shortages and drug rashes including Stevens-Johnson Syndrome, many rural Bolivians fail to complete treatment. Frequent provider interactions and direct adherence monitoring have improved outcomes in other diseases, but have not been evaluated in Chagas disease [9]. To determine the utility of pill counts and frequent monitoring to improve treatment completion and reduce adverse drug events (ADEs), we conducted a prospective cohort study of Bolivian adults treated with benznidazole for chronic *T. cruzi* infection.

## Methods

### Ethics Statement

This protocol was approved by the Institutional Review Boards of Johns Hopkins University, CEADES Salud y Medio Ambiente, the Centers for Disease Control and Prevention, and Asociación Beneficia PRISMA. Prospective cohort patients provided written informed consent prior to enrollment. No personal identifying information was extracted from the medical charts of historical patients.

### Site and Participants

Hospital Dr. Manuel Ascencio Villarroel de Punata (Punata Hospital) is a rural government referral hospital with a catchment of ∼50,000 people, located ∼45 minutes from Cochabamba, Bolivia's 3^rd^ largest city. Regional insecticide campaigns against *Triatoma infestans*, the principle *T. cruzi* vector, reduced household infestation rates from 56.6% in 1999 to 1.7% in 2004 [10].

### Study Design

**Figure 1:**
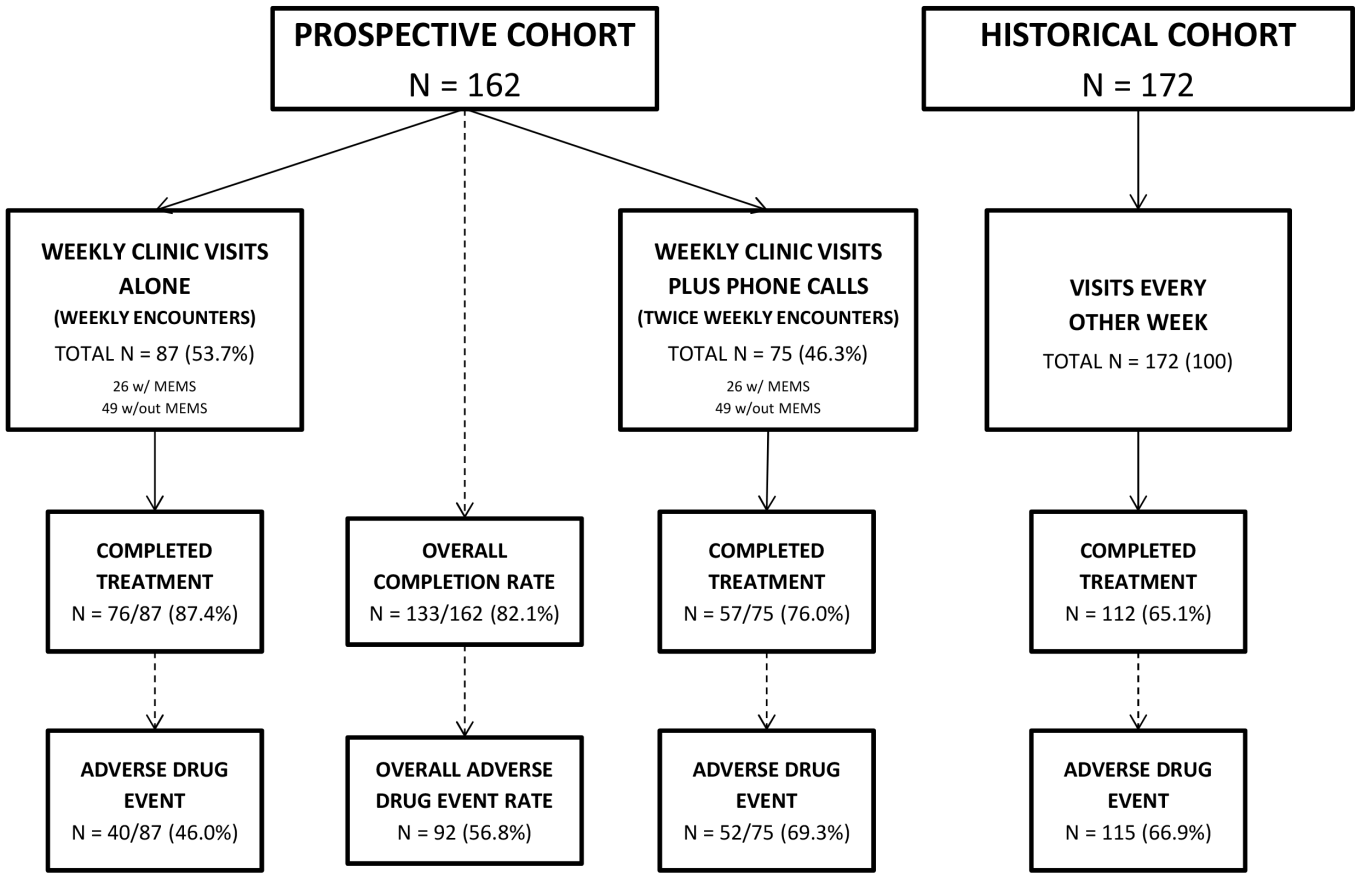
Flow of participants.

A prospective cohort was randomized to varying frequency of clinical evaluations during benznidazole treatment. The decision to initiate treatment was made by hospital physicians independent of the study, who referred patients to investigators for this monitoring protocol. After written informed consent, patients were assigned either weekly clinic visits (weekly evaluations) or weekly clinic visits plus mid-week phone calls (twice-weekly evaluations, Figure 1) by random number generator. Medication adherence and ADEs were assessed using structured questionnaires. Three patients randomized to twice-weekly evaluations had no phone, so mid-week evaluations were conducted as home visits. Patients reporting ADEs by phone were asked to report immediately to clinic. Treatment suspension and ADE management decisions were made by treating physicians, independent of study investigators. Adherent patients without complications underwent 11 in-clinic evaluations: 1 pre-treatment evaluation, 9 weekly evaluations, and 1 post-treatment evaluation. Random subsets of each group were assigned electronic pill bottles recording time and date of opening [Medication Event Monitoring Systems (MEMS), Aardex Group, Zug, Switzerland]. As we were not evaluating drug efficacy, there was no placebo group. Prospective patients were compared to a historical cohort from Punata Hospital.

### Prospective Cohort

All patients initiating benznidazole treatment at Punata Hospital from February 19, 2009–February 18, 2010 were enrolled. Chagas disease was confirmed by positive results by both *T. cruzi* lysate ELISA (Weiner laboratory, Rosario Argentina) and indirect hemagglutination assay (Lemos Laboratory, Buenos Aires, Argentina). When tests were discordant, a recombinant *T. cruzi* ELISA (Weiner laboratory, Rosario Argentina) served as tiebreaker. Inclusion criteria were age 18–60 years, residence <30 minutes from Punata Hospital, no renal, hepatic, or cardiac disease, no household vector infestation by self-report, and no upcoming travel plans. Exclusion criteria were pregnancy, residence >30 minutes from Punata Hospital, planned travel during treatment, and inability to attend clinic visits. As the area near Punata Hospital had recently been fumigated, excluding patients living >30 minutes away reduced the likelihood of vector infestation. Patients reporting active infestation were reported to the local vector control agency and became eligible for treatment after repeat fumigation. A study physician reviewed charts for pretreatment gastrointestinal disease and characterized patients by New York Heart Association (NYHA) functional classification according to standard definitions of cardiac disease without functional limitation in ordinary activity (Class I), mild dyspnea or angina with slight limitation of ordinary activity (Class II), marked limitation of ordinary activity such as walking 100 meters but no symptoms at rest (Class III), or symptoms at rest (Class IV). Pretreatment ECGs were interpreted by a cardiologist blinded to serostatus using a validated tool [11]. ECGs with heart rate <50, AV nodal block, and/or intraventricular conduction delays were considered consistent with Chagas disease [2]. Patients with symptomatic heart failure or bradycardia were referred to a cardiologist and considered eligible following appropriate treatment.

### Benznidazole Adherence Monitoring

Benznidazole was provided twice daily for 60 days at 5–7 mg/kg-day (maximum 400 mg) for 120 total doses [12]. Adherence was evaluated by self-report, weekly pill counts, and MEMS. Medications were provided weekly, with 3 extra tablets dispensed to measure over- and under-use (intentional over-distribution). Participants were blinded to pill counting and MEMS. Pill counts were reviewed during week 8, and treatment duration was extended to ensure 120 total doses. Completion was defined as ingestion of ≥114 doses (≥95% total) by pill counts. Patients who consumed 114 doses despite temporary suspension during ADEs were classified as completing treatment. MEMS data were used to validate other adherence measures. MEMS openings within 3 hours of other openings were excluded [13]. Patients who did not return as scheduled were classified as abandoning treatment.

### Adverse Drug Events

Screening consisted of 6 sequential questions: 1) “have you had any problems since your last visit,” 2) “have you had any problems with your skin,” 3) “have you had any rashes or peeling of your skin,” 4) “have you had any swelling of your hands or feet,” 5) “have you had any fevers,” and 6) “have you had any digestive problems?” Any positive answer prompted complete clinical evaluation. Each ADE had date of onset, organ system, and severity recorded in addition to prescription of analgesics, antacids, antihistamines, or anti-inflammatory drugs. Dermatologic ADEs were defined as localized rash or pruritis (“mild”), generalized rash with desquamation (“moderate”), and generalized rash involving mucosal surfaces (“severe”). Gastrointestinal ADEs were defined as intermittent discomfort (“mild”), persistent abdominal pain with nausea, vomiting, or anorexia (“moderate”), and constant pain with weight loss or hepatotoxicity (“severe”). Neurological ADEs were defined as peripheral neuropathy and persistent headaches without functional limitation (“mild”), with partial functional limitation (“moderate”), or with complete functional limitation, ageusia, or anosmia (“severe”) [14].

### Historical Cohort and Standard of Care

Charts were reviewed for all patients 18–60 years old treated for *T. cruzi* infection at Punata Hospital from June 6, 2006–July 26, 2007. Data extracted were treatment dates, age, sex, residence, weight, dosage, ADEs, completion, suspension, abandonment, and prescription of antacids, antihistamines, or other anti-inflammatory medications. Investigators categorized ADEs and ECGs as above. Standard of care was defined as evaluation of vector exposure, physical exam, laboratory analysis of kidney and liver function, pregnancy testing, and ECG [2], [12]. Patients were asked to return at 2 week intervals, without systematic provider intervention. Completion was defined by self-report.

### Statistical Analysis

Data were entered using Microsoft Access 2007 (Microsoft Corporation, Seattle, WA), then cleaned and analyzed using SPSS Version 15.0 (Apache Software, Chicago, IL). Differences between study groups were determined by χ^2^ or two-tailed t-test, as appropriate. Adherence by self-report, pill count, and MEMS were recorded as deviations from expected behavior (either missed or additional doses) since previous clinic visit. Deviations were summed weekly with Pearson correlation coefficients calculated to correlate adherence measurements between visits. Pill count data determined final completion rates. Univariate logistic regressions were constructed for prospective and historical cohorts to identify factors associated with completion and ADEs. Backwards stepwise multivariate models were constructed for variables yielding p<0.2 in univariate models. Final models accepted p<0.1. Kaplan-Meier curves were constructed for time to documentation of ADE, using log-rank tests to evaluate differences between groups.

## Results

### Prospective Cohort

#### Patient characteristics

In total, 162 patients were randomized to weekly (n = 87) or twice-weekly evaluations (n = 75, Figure 1). Patient contacts comprised 1,234 clinic visits, averaging 11 minutes 11 seconds, and 491 phone-based evaluations, averaging 2 minutes 32 seconds. Mean age was similar between groups (Table 1), though more twice-weekly than weekly patients were >50 years old (62.2% vs. 37.8%, p = 0.168). Abnormal pretreatment ECGs were more common among weekly than twice-weekly patients. ECG abnormalities were common (N = 49) including right bundle branch blocks (11), sinus bradycardia <50 (9), hemiblocks (5), left bundle branch blocks (5), and atrioventricular blocks (4). Four patients had multiple abnormalities. Specific ECG abnormalities were not different between study groups. Most patients had NYHA Class I–II (96.3%).

**Table 1:** Demographics, vector exposure, clinical characteristics, and outcome of prospective cohort patients, by weekly evaluations.

	Weekly Evaluations Only	Twice-Weekly Evaluations	P-Value
	# (Column %)	# (Column %)	
Total N	87	75	–
Female	42 (48.8)	47 (62.7)	0.078
Average Age (Years)	37.7	41.0	0.103
Age >35 Years Old	47 (54.0)	48 (64.0)	0.229
Owns Phone	71 (81.6)	67 (89.3)	0.168
Reported Frequent Alcohol Use	58 (67.4)	50 (66.7)	0.917
**Residence and Exposure to Principle Vector**			
Residence Within City of Punata	64 (73.6)	59 (78.7)	0.449
Average Years at Residence	21.6	20.0	0.529
Home Previously Infested	50 (58.1)	52 (69.3)	0.141
Average Years Since Infestation	12.4	11.1	0.559
**Pre-Treatment Clinical Characteristics**			
Cardiac Symptoms	16 (18.4)	12 (16.0)	0.688
NYHA Class>I	19 (22.1)	9 (12.0)	0.092
GI Symptoms	11 (12.6)	8 (10.7)	0.697
ECG Abnormalities	32 (38.6)	17 (23.9)	0.052
**Treatment Outcomes**			
Completed Treatment	76 (87.4)	57 (76.0)	0.039
Suspended Treatment	6 (6.9)	11 (14.7)	0.108
Abandoned Treatment	5 (5.7)	7 (9.3)	0.385

#### Treatment completion

Fifty-seven patients used MEMS (472 visits). Missed doses measured by pill count and MEMS were modestly correlated over visit intervals ≤7 days (R = 0.498, p<0.001), and highly correlated for visit intervals >7 days apart (R = 0.872, p<0.001). Self-report was poorly correlated with either MEMS or pill count data (R = 0.267 and R = 0.134, respectively). Completion was achieved by 82.1% of patients, and was significantly lower among twice-weekly patients than weekly patients (Table 1). There was a trend for more twice-weekly than weekly patients to have had treatment suspended. Abandonment rates were not significantly different between groups.

#### Adverse drug events

ADEs were detected in any organ system in 56.8% of prospective patients; 30.2% were moderate or severe (Table 2). ADEs were more commonly detected among twice-weekly than weekly patients. Rates of moderate or severe ADEs in any organ system did not differ between groups. Treatment with antihistamines and antacids was common and frequency of prescription did not differ significantly between weekly and twice-weekly patients (21.8% vs. 30.7%, p = 0.201, and 11.5% vs. 12.0%, p = 0.921, respectively). Use of antihistamines for mild dermatologic reactions was associated with a trend towards higher odds of progression to moderate severity (OR 4.000, p = 0.117). No severe dermatologic reactions occurred in this cohort. Reported alcohol ingestion was not associated with ADEs.

**Table 2:** Frequency and timing of adverse drug events by # weekly evaluations and reaction type.

	Frequency of Adverse Events			Median Onset of Adverse Events		
	1 Weekly Evaluation	2 Weekly Evaluation	P-Value	1 Weekly Evaluation	2 Weekly Evaluation	Log-Rank P-Value
	N (% of 87)	N (% of 75)		Day # (N = 87)	Day # (N = 75)	
All ADEs	40 (46.0)	52 (69.3)	0.003	12.0	10.0	0.048
All Mild ADEs	25 (28.7)	32 (42.7)	0.064	12.5	10.5	0.040
All Moderate/Severe ADEs	22 (25.3)	27 (36.0)	0.139	11.0	9.0	0.294
Dermatologic Reactions 1						
All ADEs	25 (28.7)	34 (45.3)	0.029	16.0	11.0	0.038
Mild ADEs	16 (18.4)	22 (29.3)	0.101	17.5	11.0	0.002
Moderate ADEs	11 (12.6)	16 (21.3)	0.139	15.5	11.0	0.015
Gastrointestinal Reactions						
All ADEs	25 (28.7)	30 (40.0)	0.131	10.5	14.0	0.229
Mild ADEs	12 (13.8)	20 (26.7)	0.040	8.0	21.0	0.024
Moderate/Severe ADEs^2^	15 (17.2)	17 (22.7)	0.387	22.0	22.0	0.379
Neurologic Reactions 3						
All ADEs	11 (12.6)	21 (28.0)	0.014	31.0	26.0	0.007
Mild ADEs	9 (10.30	17 (22.7)	0.033	27.0	16.0	0.016
Severe ADEs	3 (3.4)	5 (6.7)	0.346	33.0	36.0	0.172

1 No severe dermatologic reactions occurred in this cohort.

2 Only one severe gastrointestinal reaction occurred in this cohort (anorexia with weight loss). This was grouped with the moderate reactions for this analysis.

3 No moderate neurologic reactions occurred in this cohort. Mild ADEs included peripheral neuropathy with persistent that did not alter function. Severe ADEs included altered taste or smell, with or without persistent peripheral neuropathy.

Several patients had multiple systems affected. Of 162 prospective patients, 28 had both dermatologic and gastrointestinal ADEs (17.3%), 17 had dermatologic and neurological ADEs (10.5%), and 21 had gastrointestinal and neurological ADEs (13.0%). All 3 systems were affected in 12 patients (7.4%). Frequency of combined gastrointestinal and neurological ADEs was higher among twice-weekly than weekly patients (20% vs. 6.9%, p = 0.018). Dermatologic ADEs frequently presented as moderate severity (35.6%). Gastrointestinal ADEs were equally likely to present as mild or moderate severity (50.9% vs. 49.1%). Neurologic ADEs were most often mild (78.1%), though 21.9% presented with as severe symptoms including ageusia and persistent peripheral neuropathy. Severity of ADE at presentation was not different between study groups for any organ system.

**Figure 2:**
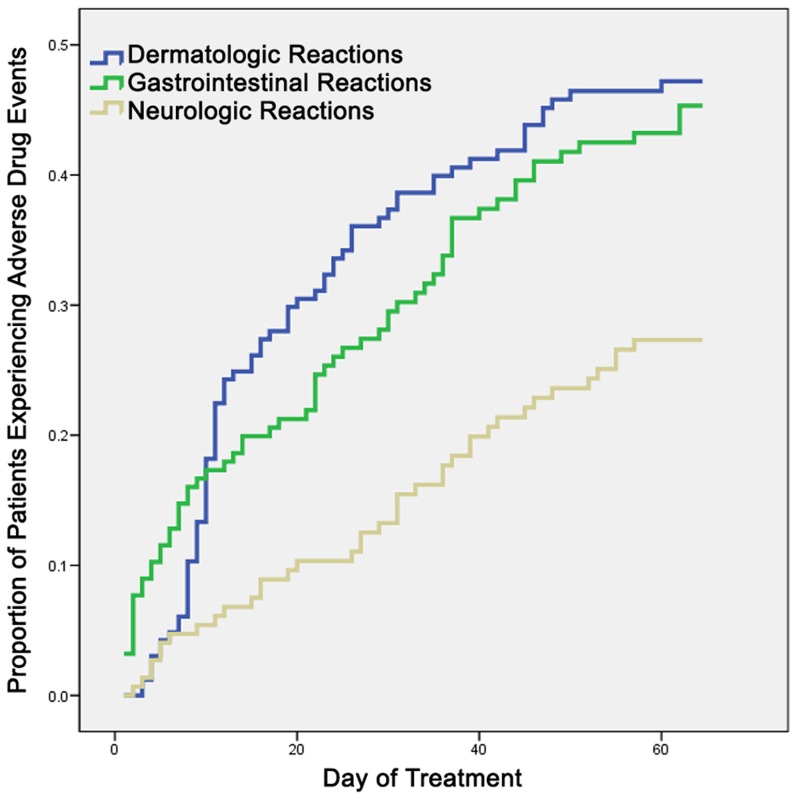
Timing of adverse drug events associated with benznidazole, by organ system.

Dermatologic ADEs occurred primarily during the 2^nd^ week of treatment, but onset occurred throughout treatment, and 18.6% were detected after the 4^th^ week (Figure 2). The earliest ADEs were gastrointestinal, but by week 2 dermatologic ADEs were more frequent. Neurologic ADEs were the least frequent throughout treatment. Mild and moderate dermatologic reactions were detected significantly earlier in twice-weekly than weekly patients (6.5 and 4.5 days earlier, respectively, Table 2).

Increased odds of ADEs were associated with female sex, twice-weekly follow-up, self-reported allergies, sun sensitivity, abnormal pretreatment ECGs, and residence within Punata rather than surrounding towns (Table 3). Increased odds of ADEs by residence persisted after controlling for total in-person encounters. ADE frequency did not vary by age.

**Table 3:** Factors associated with drug reactions, by organ system.

Univariate Analysis			Multivariate Analysis		
	Odds Ratio	P-Value		Odds Ratio	P-Value
**Any Dermatologic ADE**			**Any Dermatologic ADE**		
Twice-Weekly Evaluations	2.057	0.030	Twice-Weekly Evaluations	1.944	0.066
Female Sex	5.232	0.002	Female Sex	3.131	0.002
Abnormal ECG	1.630	0.168	Abnormal ECG	2.252	0.037
Sun Sensitivity	8.182	0.075			
**Moderate Dermatologic ADE**			**Moderate Dermatologic ADE**		
Twice-Weekly Evaluations	1.874	0.143	Female Sex	2.922	0.036
Female Sex	2.171	0.089	Residence Within City of Punata	5.048	0.040
Residence Within City of Punata	4.719	0.041	Abnormal ECG	3.905	0.005
Abnormal ECG	2.556	0.032	Sun Sensitivity	4.418	0.096
Sun Sensitivity	4.062	0.078			
**Any Gastrointestinal ADE**			**Any Gastrointestinal ADE**		
Twice-Weekly Evaluations	1.653	0.132	Female Sex	2.071	0.038
Female Sex	2.135	0.029	Sun Sensitivity	4.812	0.069
Cardiac Symptoms	1.898	0.129			
Gastrointestinal Symptoms	1.898	0.194			
Sun Sensitivity	5.200	0.054			
Family History of Allergies	5.007	0.024			
**Any Neurological ADE**			**Any Neurological ADE**		
Twice-Weekly Evaluations	2.687	0.017	Twice-Weekly Evaluations	2.832	0.015
Female Sex	1.232	0.603	Sun Sensitivity	6.961	0.018
Sun Sensitivity	6.000	0.024			
Self-Reported Allergies	1.891	0.149			
Neuropathy Before Treatment	4.913	0.121			

### Historical Cohort

#### Patient characteristics and treatment completion

Charts were reviewed for 172 patients. Prospective patients were older than historical patients, more likely to reside within Punata, and had more ECG abnormalities (Table 4). Suboptimal benznidazole dosing (<5 mg/kg-day) was documented in 49 historical and 0 prospective patients (28.5% vs. 0%, p<0.0001). Fewer historical patients completed treatment than prospective patients (65.1% vs. 82.1%, p<0.0001). Suspension rates were similar between historical and prospective cohorts (9.9 vs. 10.5%, p = 0.859), but abandonment rates were higher among historical patients (25.0% vs. 9.9%, p = 0.0003).

**Table 4:** Demographics and completion rates between prospective and historical cohorts.

	Prospective Cohort	Historical Cohort	P-Value
	# (Column %)	# (Column %)	
Total N	162	172	–
Female	89 (55.3)	103 (59.9)	0.318
Average Age (Years)	39.2	36.7	0.050
Age >35 Years Old	95 (58.6)	101 (58.7)	1.000
Residence Within City of Punata	123 (75.9)	101 (58.7)	0.004
Pre-Treatment ECG Abnormalities	49 (31.8)	22 (17.4)^1^	0.013
**Treatment Outcomes**			
Completed Treatment	133 (82.1)	112 (65.1)	<0.001
Suspended Treatment	17 (10.5)	17 (9.9)	0.859
Abandoned Treatment	12 (7.4)	43 (25.0)	<0.001

#### Adverse drug events

ADEs were more frequent among historical than prospective patients [66.9% (115/172) vs. 56.8%, p = 0.043]. Most ADEs were mild. There was no significant difference in moderate or severe ADEs of any organ system between cohorts. Prospective and historical cohorts had similar rates of antihistamine (25.9% vs. 18.0, p = 0.086) and antacid use (11.7% vs. 6.4%, p = 0.125). Patients living within Punata town were less likely to have ADEs than those living elsewhere (OR 0.427, p = 0.020). Patients >50 years old were more likely than younger patients to have moderate or severe ADEs (OR 3.333, p = 0.011). Though patients >35 years old were more likely to experience dermatologic reactions (OR 2.548, p = 0.007), age was not associated with moderate dermatologic reactions (OR 1.600, p = 0.3663). Suboptimal dosing (<5 mg/kg-day) was not associated with reduced odds of dermatologic, gastrointestinal, or neurologic ADE.

## Discussion

Enhanced follow-up employing pill-counts and phone-based encounters was associated with improved Chagas disease treatment completion, fewer ADEs, and earlier identification of ADEs. Treatment adherence is usually monitored by self-report, which poorly reflects true behavior and overestimates adherence [15]. Pill counts have been successfully employed in TB and HIV treatment, but intentional over-distribution pill counts are not widely employed [16]–[18]. Over-distribution pill counting correlated well with MEMS data, and can identify poorly-adherent patients missed by “correct distribution pill counts,” such as those who discard extra tablets [15], [19]. This study showed similar MEMS–pill count and MEMS–self-report correlations as European and American studies associating MEMS data with improved clinical outcomes [15], [19]–[21]. Improved MEMS–pill count correlation with longer inter-visit intervals suggests that over-distribution pill counts can be reliably employed over standard monitoring intervals.

Prospective patients had similar completion rates to urban Spanish and Argentinian specialty clinics (82.1% vs. 83.8% and 70%, respectively), all of whom performed better than the historical cohort [22], [23]. Phone-based controls were reliable, took under 1/4^th^ the provider time of clinic visits, and saved patients travel time. Most prospective patients had phone access (85.2%), network coverage was widely available, and cellular phone-based controls cost providers an average of $0.63, though landline-based controls were significantly less expensive. Despite similar completion rates, twice-weekly patients were more likely to complete treatment on schedule than weekly patients (data not shown), suggesting a role for phone encounters in poorly adherent patients.

ADEs were common and detected more frequently among twice-weekly than weekly patients. Overall ADE rates among prospective patients were similar to published European rates (56.8% vs. 57%), but dermatologic ADEs were less frequent (36.4% vs. 42–51%) [22], [23]. Gastrointestinal ADEs were more frequent in this study than in Spain (34.0% vs. 15%), while rates of neurologic ADEs were similar (29.8% vs. 27.6%) [22]. Moderate ADE rates were similar between prospective and historical cohorts, but were identified earlier among twice-weekly than weekly patients. This suggests that frequent encounters may over-sensitize providers to mild reactions, resulting in premature treatment suspension and lower completion rates.

Higher suspension rates among twice-weekly patients reflect the difficulty of differentiating localized (mild) from generalized, desquamating reactions (moderate) at the time of presentation. When drug rashes are identified, antihistamines should be initiated. Those with mild reactions will improve within days, and those that worsen despite antihistamines will require treatment suspension. Antihistamines did not prevent the progression of mild rashes to moderate severity, but were prescribed more frequently to patients whose reactions eventually progressed. This may be due to characteristics of greater concern to providers that were not captured by our categorization.

Predicting ADEs remains difficult. Traditional beliefs that older age, higher dose, and alcohol consumption are risk factors for severe drug reactions were not upheld by this study. Prospective and historical patients had similar rates of moderate ADEs, which were not associated with age in either cohort. Mild ADES were detected with greater frequency in the prospective cohort, especially among those with twice-weekly follow-up. There appeared to be more complete reporting of mild ADEs in older than in younger patients in the historical cohort. Suboptimal dosing was not associated with reduced ADEs in our data, consistent with new data showing no link between higher serum benznidazole concentration and ADEs [24]. Variables significantly associated with ADEs–female sex, cardiac disease, proximity to clinic, and sun sensitivity–should be accounted for in treatment and monitoring decisions. While dermatologic ADEs are classically described during the first 3 weeks, we found a continued risk throughout treatment [23]. A similar study of patients treated with nifurtimox reported ADEs in 97.5% of patients; 7.4% had severe ADEs, and only 56.2% completed treatment [8]. This study's higher completion rates and decreased frequency of ADEs confirm benznidazole as the preferred drug regimen for treatment of Chagas disease [2].

This study had several important limitations. Dedicated staff allowed clinicians to monitor ADE progression more closely than in routine practice. Completion rates may have been increased by “treating through” reactions that might otherwise have led to suspension. Exclusion of patients living >30 minutes from clinic may have also selected for prospective patients less likely to abandon treatment. Completion rates may have been affected by MEMS use, which measures bottle opening, rather than consumption. MEMS data and pill counts would ideally be compared to biomarkers or clinical outcomes, but no assay for serum benznidazole levels was available for routine use during this investigation, and no practical test of cure currently exists for chronic Chagas disease.

Conclusions from this study may not be generalizable to children. Children have fewer benznidazole-induced ADEs than adults, and older children have more frequent ADEs than younger children [25]. By restricting this study to adults, we may have missed the appropriate age-based risk categories.

Clinically, this study did not employ the Kuschnir classification or Rassi risk score [26]–[28]. These tools require results from chest radiography and echocardiography, which are not routinely employed for Chagas disease at Punata Hospital. Patients at Punata Hospital are likely similar to other rural Latin Americans, but neglecting these standardized classifications may limit comparison of our cardiac data to other populations.

Finally, the focus on treatment completion presumes a benefit that is not yet fully understood. A large, randomized trial is currently underway to evaluate the clinical efficacy of benznidazole treatment on cardiac disease, morbidity, and mortality [6]. Pending those results, this study cannot demonstrate improved long-term clinical outcomes, only improved management of patients undergoing current standard of care.

### Summary and Recommendations

Adults with Chagas disease treated with benznidazole can safely achieve high completion rates with close patient follow-up in rural Bolivia. Programs should employ over-distribution pill counts to identify poor adherence, and phone-based encounters should be considered if resources do not allow for frequent in-person evaluation. More frequent evaluations identify more ADEs but are associated with higher suspension rates. Consideration should be paid to higher odds of dermatologic ADEs among female patients, those with ECG changes, and those with prior allergies or sun sensitivity. While severe dermatologic ADEs are rare, patients must be screened for ADEs throughout the entire treatment course, not only the first few weeks. Mild side effects can be managed with antihistamines and proton pump inhibitors, but medications should not be expected to reduce ADE severity or improve completion. The most important clinical intervention for moderate or severe ADEs is withdrawal of the offending medication.
